# Development and Psychometric Validation of an App-Integrated Questionnaire to Assess Healthy Habits in Children (Ages 8–11): Implications for Pediatric Nursing Practice

**DOI:** 10.3390/children13010008

**Published:** 2025-12-19

**Authors:** María Ángeles Merino-Godoy, Carmen Yot-Domínguez, Jesús Conde-Jiménez, Emília-Isabel Martins Teixeira-da-Costa

**Affiliations:** 1 Department of Nursing, Faculty of Nursing, University of Huelva, 21071 Huelva, Spain; angeles.merino@denf.uhu.es (M.Á.M.-G.); emiliaisabel.dacosta@denf.uhu.es (E.-I.M.T.-d.-C.); 2 Didactics and Educational Organization Department, Faculty of Education Sciences, University of Seville, 41001 Seville, Spain; carmenyot@us.es; 3 Department of Theory and History of Education and Social Pedagogy, University of Seville, 41001 Seville, Spain; 4 Nursing Department, Health School, University of Algarve, 8000 Faro, Portugal; 5 Health Sciences Research Unit: Nursing (UICISA: E), Nursing School of Coimbra (ESEnfC), 3000 Coimbra, Portugal

**Keywords:** pediatric nursing, health promotion, mHealth, child health, lifestyle behaviors, psychometric validation, health literacy, school-based intervention

## Abstract

**Highlights:**

**What are the main findings?**

• A brief, four-factor questionnaire (21 items) was psychometrically validated to assess healthy habits in children aged 8–11, covering technology use, diet and growth, psychological well-being, and physical activity.
• The instrument demonstrated good model fit and reliability, supporting its use as a multidimensional assessment tool in school-aged children.

**What are the implications of the main findings?**

• The questionnaire provides pediatric nurses and educators with a practical, developmentally appropriate tool to monitor children’s lifestyle habits and identify targets for early preventive intervention. When integrated into the Healthy Jeart mHealth app, it supports feasible school-based health promotion through structured monitoring, feedback, and reinforcement of healthy behaviors.

**Abstract:**

Introduction: Promoting healthy habits in childhood is fundamental for fostering long-term well-being. This study aimed to develop and psychometrically validate an app-integrated instrument to assess knowledge, habits, and attitudes related to health in children aged 8–11, within the context of the MHealth intervention Healthy Jeart. Methods: A quantitative, cross-sectional design was used. An initial item pool underwent expert content validation before being administered to a sample of 623 children from primary education centers in Andalusia, Spain. Construct validity was examined through exploratory and confirmatory factor analyses. Results: The analyses supported a coherent four-factor structure comprising 21 items: (1) Use of technologies, (2) diet and growth, (3) psychological well-being, and (4) physical activity and well-being. The instrument demonstrated satisfactory model fit and internal consistency, providing a multidimensional assessment of children’s health-related behaviors. The sample was recruited from primary schools in Andalusia (Spain), which may limit the generalizability of the findings to other regions and cultural contexts. Conclusions: The validated instrument offers a reliable and efficient means of evaluating healthy habits in children aged 8–11, particularly when embedded within digital interventions such as Healthy Jeart. It represents a valuable tool for educators and pediatric nursing professionals working in school settings, enabling early identification of gaps in health literacy and supporting targeted interventions that promote holistic child well-being.

## 1. Introduction

Improving the health and well-being of children and adolescents within school environments has been identified as an urgent global priority [1]. This work aligns with the United Nations Sustainable Development Goals (SDGs), particularly SDG3 on Health and Well-being, which reinforces the importance of early intervention and health promotion strategies [2]. The World Health Organization [3] emphasizes that adolescence is one of the most rapid and formative stages of human development, involving multiple dimensions: physical, cognitive, social, emotional, and sexual, that require targeted attention in health and education policies. Investments in adolescent well-being generate long-term health and economic benefits, reinforcing human capital development and yielding a “triple dividend” across present and future generations [3].

Promoting healthy lifestyle habits, including a balanced diet, regular physical activity, and responsible use of technology, is essential for both somatic and emotional outcomes. Evidence shows that these behaviors are associated with improved emotional regulation, reduced anxiety, and better social functioning [4,5]. These relationships are particularly relevant within pediatric nursing practice, where nurses in school and community settings work closely with children, supporting healthy development through behavioral guidance, health education, and early risk detection. Pediatric and child mental-health nurses are central to addressing both emotional needs and lifestyle patterns that influence mental health trajectories [6]. Their proximity to children within the school environment enables direct contact, trust-building, and consistent follow-up, creating a privileged setting for interventions that integrate healthy habit formation with psychosocial support.

Digital health solutions have been increasingly incorporated into child-centered health promotion. The WHO highlights the value of mobile health (mHealth) technologies for their usability, reach, and acceptability, demonstrating effects on awareness, behavioral change, and risk reduction related to physical inactivity and unhealthy diet [7,8]. Research shows that mobile apps can promote healthier eating, increase physical activity, and reduce sedentary behavior among children and adolescents [9,10,11]. Systematic reviews confirm the efficacy of app-based interventions in improving diet and reducing inactivity [12,13], supporting the growing relevance of mHealth in school-based nursing practice and health promotion.

In Spain, rising rates of obesity and overweight among children have been documented [14,15], and unhealthy lifestyle patterns, including sedentary behavior and excessive screen time, have been observed across school-aged populations [16,17,18,19]. As a response, educational and digital interventions have been developed to promote healthier behaviors [20,21,22,23].

The availability of validated instruments to assess health-related behaviors in children remains limited. Existing tools often focus on specific domains such as diet, physical activity, sedentary behavior, or health-related quality of life (HRQoL) [24,25,26,27,28] and do not provide a brief, multidimensional evaluation suitable for younger age groups. In particular, many instruments were developed for adolescents or older children and may not be developmentally or linguistically appropriate for children aged 8–11, whose cognitive and self-reporting capacities require simplified, concrete item formulations. Moreover, cross-cultural applicability cannot be assumed, as instruments typically require systematic cultural adaptation and re-validation before use in different contexts. Additionally, studies indicate that while children aged 6–10 may possess theoretical knowledge about healthy habits, they often fail to implement them consistently in daily life [29], underscoring the need for supportive, developmentally appropriate assessment tools integrated into educational contexts where pediatric nurses and teachers collaborate.

The development of the assessment instrument presented in this study should be understood as a theory-driven and staged process that precedes and supports its psychometric validation. This process is built upon previous phases of the Healthy Jeart research program, in which the conceptual framework, content domains, and educational foundations of the app were established and empirically explored in adolescent populations through qualitative methods, expert consensus, and intervention-based studies [30,31]. These earlier phases provided a robust theoretical basis for defining multidimensional health-related constructs and translating them into educational content and assessment indicators suitable for use in school settings. The present study represents a subsequent step in this research trajectory, focused on adapting and operationalizing this established framework for children aged 8–11 years.

The conceptual framework underlying the instrument comprises several interrelated dimensions reflecting a holistic and preventive approach to child health, consistent with international health-promotion models [3,7]. The use of technologies dimension addresses responsible and supervised engagement with digital devices and awareness of health risks associated with excessive screen exposure and unsafe online interactions [7,8]. The diet and growth dimension focuses on nutritional literacy and everyday eating behaviors, including understanding of the food pyramid, adherence to the Mediterranean diet, and informed food choices [4,12]. The remaining dimensions, physical activity and physical well-being and psychological well-being, are conceptually distinct but complementary, capturing behaviors related to physical functioning and socio-emotional adaptation, respectively, while maintaining clear boundaries between physical and psychological domains [3,5,9]. Psychological well-being in childhood has been conceptualized from multiple theoretical perspectives, including positive psychology, developmental psychology, and health-related quality-of-life research [24,25,26]. Within health promotion frameworks, it is understood not as the absence of mental disorders, but as the presence of positive emotional states, adaptive coping strategies, and socio-emotional skills that support healthy development and social participation [3,24]. In this study, psychological well-being is operationalized in functional and developmentally appropriate terms, focusing on observable attitudes and behaviors that can be reliably self-reported by children aged 8–11 years, such as empathy and perspective-taking, positive emotional orientation, constructive problem-solving, and help-seeking behaviors. This approach deliberately avoids clinical or diagnostic interpretations and aligns with preventive and educational models of child well-being, as well as with the conceptualization adopted in earlier phases of the Healthy Jeart research program [25,26,27,28,30,31].

### Healthy Jeart App

**Figure 1:**
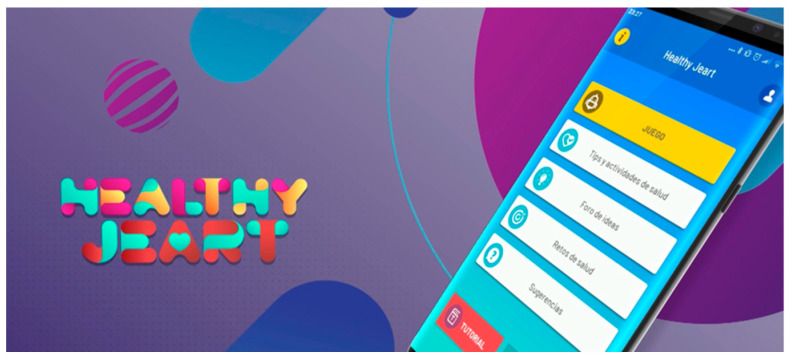
Screenshot of the main screen of Healthy Jeart.

The Healthy Jeart app (Figure 1) was designed using qualitative and interdisciplinary methods [32,33] to promote healthy lifestyles among children aged 8–16. It integrates educational content across different topics, interactive elements, and classroom-compatible challenges [34,35]. The app was formally certified as a Healthy App by the Agency of Health Quality of Andalusia in 2018, confirming its reliability and safety.

Healthy Jeart incorporates an assessment tool for children aged 8–11, enabling tracking of knowledge, attitudes, and habits related to five dimensions: nutrition, psychological well-being, physical activity, physical well-being and responsible technology use. Importantly, this tool serves not only as a digital monitor but also as a practical clinical-educational instrument that pediatric nurses can use to identify risk factors, plan interventions, and support healthy habit formation among children in school settings.

This study aimed to develop and psychometrically validate this app-integrated assessment tool. By linking everyday habits to indicators of physical and psychological well-being, it provides pediatric nurses, educators, and mental-health professionals with a holistic, evidence-based method for monitoring early health behaviors and promoting child well-being.

## 2. Methods

### 2.1. Aim

The primary objective of this study was to develop and psychometrically validate an app-integrated instrument within the Healthy Jeart platform to assess health-related knowledge, habits, and attitudes among children aged 8–11.

### 2.2. Design

A quantitative, cross-sectional validation design was used, comprising two sequential phases: (1) expert-based content validation of the initial instrument, followed by (2) empirical construct validation with the target population. Ethical approval was obtained from the Research Ethics Committee of the Province of Huelva, in accordance with the principles of the Declaration of Helsinki. Participation was voluntary, parental informed consent was required, and all data were handled in compliance with applicable Spanish and European data protection regulations (GDPR).

### 2.3. Instrument Development and Expert Validation

The initial version of the instrument consisted of 56 items distributed across five conceptual domains: physical activity (9 items), nutrition (12 items), physical well-being (10 items), psychological well-being (15 items), and new technologies (10 items). These items were formulated based on educational content embedded in the Healthy Jeart app.

A panel of 15 experts, including primary school educators and specialists in child health promotion, evaluated the items using a ten-point scale to assess clarity, internal coherence, responsiveness, appropriateness of language, and construct measurement accuracy. Panelists also assessed overall instrument clarity, linguistic adequacy for children aged 8–11, and domain organization. Recommendations included wording modifications and the addition of one item to the physical activity domain. Following this refinement, the instrument was reduced to 22 items, which scored an average of over 8 in each evaluated aspect. Given that the expert panel included more than two evaluators, inter-rater agreement was not assessed using kappa statistics. Instead, a quantitative consensus criterion was applied: items were retained when they achieved a mean score > 8 (on a 10-point scale) across all evaluated dimensions (clarity, internal coherence, responsiveness, appropriateness of language, and construct measurement accuracy), ensuring a high level of agreement and supporting the content validity of the instrument.

### 2.4. Participants and Data Collection

A convenience sample was recruited from seven educational centers located in Andalusia, Spain: three in Córdoba, one in Huelva, and three in Seville. Participants were students enrolled in Year 4 through Year 7 of Primary Education, corresponding to ages 8–11.

Data collection occurred between November 2022 and March 2023. The final sample consisted of 623 children (308 boys, 315 girls). Of these, 245 participants were aged 8–9 and 378 were aged 10–11. The distribution by school year was as follows: Year 4 = 22.63%, Year 5 = 17.5%, Year 6 = 35.31%, and Year 7 = 24.56%.

The instrument was administered in paper format during classroom sessions. Instructions were provided in person by the classroom teacher and/or a member of the research team. Children completed the questionnaire individually and anonymously, without interaction or discussion.

### 2.5. Data Analysis

Data was analyzed using JASP (version 0.11.1.0) and SPSS (version 26). Initially, descriptive statistics were calculated, including means, standard deviations, ranges, and minimum and maximum values. Principal Component Analysis (PCA) with ProMax oblique rotation was conducted to estimate the underlying factor structure and identify items with factor loadings above 0.40. Promax oblique rotation was selected because correlated dimensions were theoretically expected, given the interrelated nature of healthy lifestyle behaviors in childhood

Parallel analysis was used to determine the number of extracted components. Subsequently, Exploratory Factor Analysis (EFA) was performed to evaluate preliminary model fit, which demonstrated acceptable indices (RMSEA = 0.023; TLI = 0.926). Items adversely affecting fit indices were candidates for removal.

Confirmatory Factor Analysis (CFA) was then applied to test the final factor structure. Model adequacy was assessed using standard fit criteria [36,37], where CFI and GFI ≥ 0.96, TLI ≥ 0.95, and RMSEA ≤ 0.05 indicate excellent fit. Values of CFI, GFI, TLI ≥ 0.90 and RMSEA ≤ 0.08 indicate acceptable fit. SRMR ≤ 0.08 was also taken as evidence of a good fit [38]. Internal consistency was evaluated using Cronbach’s alpha and McDonald’s omega. Measurement invariance testing (e.g., by gender or age subgroups) and cross-validation were not performed, as the primary aim of this study was to establish the factorial structure and psychometric properties of the instrument in children aged 8–11 years. These analyses are planned for future research with more diverse samples and broader study aims. Moreover, previous Healthy Jeart research has examined distinct developmental groups, and the adolescent version has been validated separately [30,31].

## 3. Results

The factor structure identified through PCA and subsequently confirmed through CFA supported a four-factor solution comprising 21 items. These factors were conceptually aligned with the dimensions of health targeted by the Healthy Jeart intervention: (1) Use of technologies, (2) Diet and growth, (3) Psychological well-being, and (4) Physical activity and physical well-being. This multidimensional structure reflects the holistic nature of healthy lifestyle development in children. The primary fit indices for the four-factor model are presented in Table 1. Although chi-square-based statistics were considered, they were not emphasized due to their sensitivity to sample size; therefore, model evaluation prioritized incremental and absolute fit indices commonly recommended for latent variable models. The results showed a CFI of 0.908 and IFI of 0.911, both above the recommended threshold of 0.90, indicating acceptable comparative fit. The TLI value of 0.885 falls within the range typically described as marginally acceptable in multidimensional behavioral models with heterogeneous item content. Although the NFI (0.809) and RFI (0.761) were lower, as expected in multifactorial pediatric self-report instruments, the PNFI value of 0.647 supports the parsimony and practical applicability of the final model in time-constrained school-based assessment settings.

Additional fit indices further supported the adequacy of the model (Table 2). The RMSEA value of 0.034, with a narrow 90% confidence interval (0.027–0.040), indicates a close fit of the model to the data, well below the commonly accepted threshold of 0.05 for good model fit. The very high GFI of 0.997 demonstrates an excellent level of explained variance in the observed covariance matrix, while the SRMR of 0.043 falls below the recommended cutoff of 0.08, indicating low average residuals between observed and predicted correlations. Together, these indices provide strong evidence of the model’s structural robustness and support the stability of the four-factor structure for pediatric behavioral assessment.

Factor loadings ranged from moderate to strong across items (Table 3). Several items showed substantial loadings above 0.60, including NT00006 (0.912), BF00006 (0.734), and ALM00010 (0.892), indicating strong contributions to their respective latent constructs. Most item loadings were statistically significant (*p* < 0.001), with confidence intervals that did not cross zero, supporting the discriminant validity of the items. By contrast, item ALM00008 presented a weaker loading (0.037) and a confidence interval crossing zero, suggesting lower item-construct association and possible conceptual redundancy within the Diet and growth factor. Despite this, ALM00008 was retained for content validity reasons, which are further discussed in Section 4.

Item-level residual variances are presented in Table 4. All items showed statistically significant residual estimates (*p* < 0.001) with positive confidence intervals, indicating stable and non-random residual variability. Several items, such as NT00007 (residual = 1.881) and ALM00012 (1.943), exhibited higher residual values, suggesting greater unexplained variance, which may reflect the inherent complexity of behavioral constructs such as digital engagement and nutritional understanding in children. Conversely, items with lower residual variances, including BP00010 (0.601) and BP00013 (0.754), indicate tighter alignment with their latent factors, particularly within the psychological well-being domain. These results support the overall contribution of each item to the model, while reflecting expected variability within a pediatric self-report instrument measuring multidimensional lifestyle behaviors.

Lastly, mean score analysis provided further insight into the behavioral patterns assessed. Within the “Use of technologies” domain, children reported generally responsible and mindful digital practices, with all items scoring above 3.0 and “I make a responsible use of my mobile phone, tablet, etc.” showing the highest endorsement (M = 4.09, SD = 1.12). In contrast, responses within “Diet and growth” revealed a discrepancy between perceived healthy food consumption and actual nutritional literacy. While children strongly affirmed adherence to Mediterranean diet principles, as reflected in “Every day, I consume foods from the Mediterranean diet (olive oil, tomatoes, fish, cereals…)” (M = 4.34, SD = 0.99), their knowledge of appropriate nutritional portions was less robust, evidenced by the much lower score for “I know the amount of food I must consume every day according to the food pyramid” (M = 2.804, SD = 1.474). Items in the “Psychological well-being” factor consistently obtained means above 4.0, with strong endorsement of empathy-related behaviors such as “I consider that putting myself in other people’s shoes is important when interacting with them” (M = 4.401, SD = 0.814), suggesting notable socioemotional competence. Similarly, most items in “Physical activity and well-being” reached scores above 4.0, indicating strong engagement in movement and exercise. However, children reported suboptimal hydration habits, with the item “I drink at least 2 L of water every day” yielding a lower mean (M = 3.334, SD = 1.449), highlighting a lifestyle aspect requiring improvement.

## 4. Discussion

The results of this study provide robust evidence for the psychometric validity of the tool integrated within the Healthy Jeart app, designed to assess health-related knowledge, habits, and attitudes in children aged 8–11. The validated structure, comprising four key behavioral domains (use of technologies, diet and growth, psychological well-being, and physical activity and well-being), reflects an integrative model of pediatric health that acknowledges the interplay of physical, emotional, nutritional, and behavioral dimensions in early development [6,39,40].

The validation process indicated that the instrument successfully captures distinct aspects of child behavior without redundancy, as demonstrated by the reduction of items from 56 to 22 following expert review. This refinement ensured that each item remained conceptually relevant and statistically informative, allowing for a more efficient and meaningful assessment of children’s lifestyles. The confirmatory factor analysis sustained the four-domain structure (21 items), confirming that the tool reliably represents the intended latent constructs and reinforcing its suitability for systematic use in educational and clinical settings. Compared with many existing pediatric assessment tools that focus on single behavioral domains or broader quality-of-life constructs, this questionnaire offers a brief, multidimensional evaluation specifically designed for school-aged children and preventive practice.

The integration of this validated tool into a mobile-health platform extends its practical value. The Healthy Jeart app facilitates continuous monitoring and self-reflection, enabling children to engage actively with their own health behaviors. The ability of the app to provide users with feedback and reinforce positive habits promotes meaningful engagement, consistent with the demonstrated success of digital interventions in youth health promotion [12]. Such functionality is particularly relevant in modern health education, where mobile technologies increasingly mediate children’s learning and daily routines.

Importantly, the tool’s multidimensional structure provides actionable insights into specific behavioral patterns. The strong performance of items in the psychological well-being domain suggests that empathy, emotional regulation, and socio-emotional understanding are already well internalized at this age, aligning with evolving curricular emphases on emotional intelligence in schools [4,5]. Conversely, lower nutritional knowledge observed in items related to portion quantity indicates a need for targeted interventions to strengthen nutrition literacy. Regarding item ALM00008, its low factor loading suggests a weaker statistical association with the latent construct. However, the item was retained due to its high expert ratings and its relevance for assessing dietary literacy within the Mediterranean cultural context. Retaining this item supports content validity and alignment with regionally appropriate nutritional guidance, although its psychometric performance should be further examined in future studies with larger and more diverse samples. These findings echo previous research showing that children often recognize “healthy” foods but lack an accurate understanding of dietary recommendations [16,29]. These findings underscore the potential role of school-based pediatric and mental health nurses, who can leverage the tool as part of preventive monitoring and early intervention strategies. By identifying gaps in nutritional education or hydration habits, and by reinforcing healthy digital behavior and physical activity, nurses are well positioned to support holistic child development. The tool thus contributes to bridging general preventive health strategies with more targeted mental well-being initiatives, reflecting evolving models of integrated pediatric care. In this sense, the questionnaire may support structured screening, monitoring, and tailored health education activities within school health services, facilitating collaboration between nurses, teachers, and families.

Finally, while the tool is delivered through a digital platform, the potential risk of increased screen exposure must be carefully managed. Its use should be embedded within structured, time-limited educational activities and combined with offline tasks that reinforce physical activity, family interaction, and real-world behavior change. This approach is consistent with current recommendations emphasizing controlled and purposeful screen use to protect children’s physical and psychological well-being [41,42,43]. Although Healthy Jeart already incorporates features such as offline activity prompts and screen-free intervals, their effectiveness ultimately depends on coordinated reinforcement by parents, educators, and health professionals within school and family environments.

### Limitations and Future Research

This study has several limitations that should be considered when interpreting the findings. First, the sample was obtained through convenience sampling from primary schools in Andalusia, which may limit the generalizability of the results to wider child populations. As validation was conducted within a specific Spanish regional context, cross-cultural transferability cannot be assumed, and future studies should address systematic translation, cultural adaptation, and re-validation in other settings. Future research should also include more diverse samples to confirm the stability of the four-factor structure across different cultural and socio-economic contexts. Where feasible, stratified or probability-based sampling strategies are recommended to strengthen representativeness.

Second, the cross-sectional design did not allow examination of temporal stability. Longitudinal research, including test-retest analyses, is needed to assess the instrument’s reliability over time and its sensitivity to changes in children’s health behaviors.

Additionally, the present study was designed to develop and validate the instrument rather than to test group differences. Associations between instrument scores and demographic variables (e.g., gender or age subgroups) were therefore beyond the scope of this manuscript and should be addressed in future studies focused on behavioral profiling and subgroup comparisons.

Importantly, the usability and acceptability of the Healthy Jeart app have already been evaluated in previously published research [44], demonstrating strong user satisfaction and feasibility in school settings. Therefore, usability is not considered a limitation of the present study. Future work should instead explore how the validated instrument performs when used longitudinally and across different educational or cultural contexts. As the tool is delivered through a digital platform, continued attention is needed to ensure that its implementation supports learning without contributing to excessive screen time.

Finally, further research should examine the instrument’s predictive validity and its adaptability across additional languages and health-promotion programs, strengthening its applicability as a brief, multidimensional measure for monitoring healthy habits in childhood.

## 5. Conclusions

This study presents a validated and multidimensional tool integrated within the Healthy Jeart app to assess health-related knowledge, attitudes, and habits in children aged 8–11. The instrument demonstrated strong psychometric performance and captured behavioral constructs that are essential for early health promotion, including digital behavior, nutritional literacy, psychological well-being, and physical activity.

By combining a rigorous psychometric foundation with digital deployment through a user-friendly mHealth platform, the Healthy Jeart tool represents a valuable addition to school-based health education and pediatric behavioral assessment. The ability to monitor children’s habits in real time and provide tailored feedback offers new opportunities for early preventive action, particularly for pediatric and psychiatric-mental health nurses working in educational settings. In practice, the tool can be integrated into routine school health services through coordinated use by pediatric nurses and teachers, supported by brief training and structured implementation guidelines. Engagement of families through feedback mechanisms and home-based activities may further enhance consistency and impact in promoting healthy habits.

The findings highlight the potential of digital assessment tools to support equitable access to health literacy and habit formation, in alignment with global priorities for early intervention and child well-being. Moreover, the tool supports a holistic understanding of childhood lifestyles, enabling targeted interventions in areas such as nutrition and hydration while reinforcing positive competencies like empathy and responsible technology use.

Continued refinement and scaling of the Healthy Jeart tool, along with further investigations into its longitudinal effects and adaptability across diverse populations, will be essential for maximizing its impact. Ultimately, this validated instrument contributes to a growing ecosystem of digital health strategies designed to empower children as active participants in developing their own lifelong healthy habits.

## Figures and Tables

**Table 1:** Comparative Fit Indices of the Four-Factor Model.

Index	Value
Comparative Fit Index (CFI)	0.908
Tucker–Lewis Index (TLI)	0.885
Bentler–Bonett Non-normed Fit Index (NNFI)	0.885
Bentler–Bonett Normed Fit Index (NFI)	0.809
Parsimony Normed Fit Index (PNFI)	0.647
Bollen’s Relative Fit Index (RFI)	0.761
Bollen’s Incremental Fit Index (IFI)	0.911
Relative Noncentrality Index (RNI)	0.908

CFI, IFI, and RNI ≥ 0.90 indicate acceptable model fit; TLI values between 0.85 and 0.90 are considered marginally acceptable in multidimensional behavioral models with child self-report data [36,38].

**Table 2:** Absolute and Residual Fit Indices of the Four-Factor Model.

Metric	Value
Root mean square error of approximation (RMSEA)	0.034
RMSEA 90% CI lower bound	0.027
RMSEA 90% CI upper bound	0.040
RMSEA *p*-value	1.000
Standardized root mean square residual (SRMR)	0.043
Hoelter’s critical N (α = 0.05)	433.837
Hoelter’s critical N (α = 0.01)	464.933
Goodness of fit index (GFI)	0.997
McDonald fit index (MFI)	0.909
Expected cross-validation index (ECVI)	0.730

RMSEA ≤ 0.05 and SRMR ≤ 0.08 indicate good approximate model fit; GFI ≥ 0.95 reflects strong variance explanation by the model [36,38].

**Table 3:** Standardized Factor Loadings for Each Item.

							95% Confidence Interval	
Factor	Item Code	Symbol	Estimate	Std. Error	z-Value	*p*	Lower	Upper
Factor 1	BF00006	λ11	0.734	0.065	11.340	<0.001	0.607	0.861
	NT00006	λ12	0.912	0.065	14.012	<0.001	0.785	1.040
	NT00007	λ13	0.695	0.067	10.318	<0.001	0.563	0.827
	NT00003	λ14	0.487	0.049	9.930	<0.001	0.391	0.584
Factor 2	ALM00002	λ21	0.263	0.053	4.956	<0.001	0.159	0.367
	ALM00006	λ22	0.227	0.049	4.637	<0.001	0.131	0.323
	ALM00012	λ23	0.477	0.070	6.769	<0.001	0.339	0.615
	ALM00008	λ24	0.037	0.051	0.717	0.473	−0.064	0.137
	ALM00001	λ25	0.376	0.050	7.568	<0.001	0.278	0.473
	ALM00004	λ26	0.406	0.059	6.925	<0.001	0.291	0.522
	ALM00010	λ27	0.892	0.077	11.564	<0.001	0.741	1.043
Factor 3	BP00010	λ31	0.245	0.041	5.951	<0.001	0.165	0.326
	BP00013	λ32	0.325	0.047	6.931	<0.001	0.233	0.417
	BP00006	λ33	0.594	0.057	10.438	<0.001	0.483	0.706
	BP00008	λ34	0.342	0.048	7.083	<0.001	0.248	0.437
	BP00007	λ35	0.486	0.058	8.303	<0.001	0.371	0.600
Factor 4	AF00009	λ41	0.206	0.053	3.894	<0.001	0.102	0.309
	AF00008	λ42	0.228	0.048	4.761	<0.001	0.134	0.322
	AF00004	λ43	0.184	0.044	4.207	<0.001	0.098	0.270
	AF00007	λ44	0.232	0.048	4.870	<0.001	0.138	0.325
	BF00005	λ45	1.056	0.097	10.928	<0.001	0.866	1.245

Item wording corresponding to item codes is provided in Appendix A. λij denotes the standardized loading of item j on factor i.

**Table 4:** Residual Variances of Individual Items.

					95% Confidence Interval	
Indicator	Estimate	Std. Error	z-Value	*p*	Lower	Upper
BF00006	1.674	0.106	15.735	<0.001	1.465	1.882
NT00006	1.434	0.104	13.772	<0.001	1.230	1.638
NT00007	1.881	0.116	16.165	<0.001	1.653	2.109
NT00003	1.014	0.062	16.306	<0.001	0.893	1.136
ALM00002	1.100	0.064	17.084	<0.001	0.974	1.227
ALM00006	0.978	0.057	17.206	<0.001	0.867	1.090
ALM00012	1.943	0.117	16.637	<0.001	1.714	2.172
ALM00008	0.980	0.055	17.686	<0.001	0.871	1.088
ALM00001	0.937	0.057	16.326	<0.001	0.825	1.050
ALM00004	0.887	0.061	14.434	<0.001	0.767	1.007
ALM00010	1.190	0.124	9.601	<0.001	0.947	1.433
BP00010	0.601	0.036	16.506	<0.001	0.529	0.672
BP00013	0.754	0.047	16.041	<0.001	0.662	0.846
BP00006	0.878	0.068	12.861	<0.001	0.744	1.011
BP00008	0.795	0.050	15.959	<0.001	0.697	0.892
BP00007	1.103	0.073	15.171	<0.001	0.960	1.245
AF00009	1.151	0.066	17.364	<0.001	1.021	1.281
AF00008	0.956	0.056	17.213	<0.001	0.847	1.065
AF00004	0.799	0.046	17.310	<0.001	0.709	0.890
AF00007	0.940	0.055	17.192	<0.001	0.833	1.047
BF00005	0.983	0.184	5.352	<0.001	0.623	1.342

All residuals were statistically significant at *p* < 0.001. Full item wording is available in Appendix A.

## Data Availability

The data presented in this study is available upon request from the corresponding author. The data are not publicly available due to privacy and ethical reasons.

## Appendix A

The table below presents the English translation of the Healthy Habits Questionnaire originally validated in Spanish. The items are shown by factor and include the original item codes used during instrument development. The Spanish version was used for data collection, while the English version is provided here for transparency and international readability

**Table A1:** English Translation of the Original Spanish Healthy Habits Questionnaire.

Factor 1—Use of technologies	BF00006	I avoid high volumes in my headphones and bright screens in my mobile phone, tablet, etc., because it is bad for my health.
	NT00006	I let my family see how I use my mobile phone, tablet, game console, etc.
	NT00007	I speak with my family (father, mother, siblings…) about the contacts and conversations I have in my social networks.
	NT00003	I make a responsible use of my mobile phone, tablet, etc.
Factor 2—Diet and growth	ALM00002	I know the amount of food I must consume every day according to the food pyramid.
	ALM00006	I try to have a healthy, varied and balanced diet.
	ALM00012	I search for reliable information to eat healthy and, therefore, better.
	ALM00008	Every day, I consume foods from the Mediterranean diet (olive oil, tomatoes, fish, cereals…).
	ALM00001	I distinguish the types of foods in the food pyramid.
	ALM00004	I can differentiate which foods have fats that are bad for my health (e.g., milk chocolate, packaged potato chips, butter…) and which foods have healthy fats (nuts, corn, avocado, sardines…).
	ALM00010	I avoid sweet foods such as sweets, pastries, etc.
Factor 3—Psychological well-being	BP00010	I consider that putting myself in other people’s shoes is important when interacting with them.
	BP00013	Every day, I think of things that make me happy (family, food, friends, etc.).
	BP00006	I think that problems with other people (friends, relatives, etc.) are opportunities to learn to solve them and improve.
	BP00008	I know that if I am more positive toward life, I will have fewer problems.
	BP00007	I ask my friends and relatives for help to overcome my problems.
Factor 4—Physical activity and physical well-being	AF00009	I believe that performing physical activity (e.g., walking, swimming, etc.) helps me to feel better and be in a better mood.
	AF00008	I drink water when I do sports because I know it is good for my health.
	AF00004	I try to perform physical activity (running, hiking, etc.) outdoors with my friends and relatives (siblings, cousins, parents…).
	AF00007	It is important to warm up and stretch before and after performing physical activity (e.g., hiking, dancing, etc.).
	BF00005	I drink at least 2 litres of water every day.

Note. Items are presented in English for publication purposes. The instrument was validated and applied in its original Spanish version. Item codes correspond to the original item bank. All items were rated on a 1–5-point Likert scale (1 = strongly disagree; 5 = strongly agree).
